# Integrative multi-omics reveal the impact of different light spectra on lateral branching and growth of medicinal plant *Artemisia argyi*

**DOI:** 10.3389/fpls.2026.1871654

**Published:** 2026-10-12

**Authors:** Ahad Mehmood, Zhao Yipeng, Jiali Song, Zhenchao Yang, Yongjun Wu

**Affiliations:** 1 College of Life Sciences, Northwest A&F University, Yangling, China; 2 Key Laboratory of Northwest Facility Horticulture Engineering of Ministry of Agriculture and Rural Affairs, College of Horticulture, Northwest A&F University, Yangling, China

**Keywords:** *Artemisia argyi*, medicinal plants, physiological traits, RNA sequencing and molecular pathways, supplemental LEDs light

## Abstract

Light quality is a decisive environmental regulator of growth and secondary metabolism in medicinal plants, yet the spectral requirements of *Artemisia argyi*, a species prized for bioactive metabolites such as artemisinin, eupatilin, and jaceosidin, remain poorly defined. This study integrated physiological, transcriptomic, and metabolomic analyses to evaluate the response of *A. argyi* to 4 weeks of supplemental light-emitting diode (LED) lighting of three spectral compositions: blue–red (BR, 3:1), red–blue (RB, 3:1), and white (control), with three biological replicates per treatment. Physiologically, BR light produced the tallest stems, the largest and densest foliage, and the greatest stem diameter, shoot fresh and dry weight, and number of lateral branches; relative to RB and white light, plant height increased by 109%, 100%, 78%, and 8.6% and leaf number by 51.61%, 58.33%, 37.20%, and 4.2% across weeks 1–4, accompanied by higher antioxidant enzyme activity and reactive oxygen species (ROS) levels. RNA sequencing yielded 44.18–50.75 million raw reads per sample with a mapping ratio of 83.47%–87.06% and Q30 > 94%. Pairwise analysis identified 2,025 differentially expressed genes (DEGs; 1,173 upregulated and 852 downregulated) for BR vs. RB, 974 (652 upregulated and 322 downregulated) for BR vs. white, and 601 (141 upregulated and 460 downregulated) for RB vs. white, enriched in light-responsive, photosystem, chlorophyll- and proline-metabolism and circadian pathways. Key regulators included the two-component response regulator-like APRR1 (a CheY-like phosphoacceptor), proline dehydrogenase (ProDH), the chlorophyll b reductase NOL, and terpenoid-backbone (isoprene) genes. Metabolomic profiling detected 174, 129, and 81 differentially accumulated metabolites in BR vs. RB, BR vs. white, and RB vs. white, respectively, with 486 metabolites up- and 459 downregulated in BR vs. RB and pronounced enrichment of amino acid, carbohydrate, lipid, and secondary-metabolite (flavonoid, terpenoid, and alkaloid) biosynthesis. Collectively, these results demonstrate that BR supplemental light coordinately enhances growth, lateral branching, and bioactive-compound biosynthesis, offering a practical basis for the precision cultivation of *A. argyi* and the targeted production of its medicinal compounds.

## Introduction

*Artemisia argyi* (mugwort) has been employed in traditional Chinese medicine for more than 2,000 years to treat various diseases, including fever, hemorrhoids, and diseases associated with fever. Aromatic wreaths, spirits, and the perfume industry utilize it. Artemisinin, the principal bioactive compound, exhibits antifungal, antibacterial, and periodontopathic properties (Cui et al., 2023; Zhang et al., 2023). *A. argyi*, a perennial herb medicinal application, extends in treating different disease conditions such as irregular menstruation, menorrhagia, metrorrhagia, dermatitis, dysmenorrhea, and pruritus (Hu et al., 2024; Zhang et al., 2023). The plant is extensively utilized in its original form, with applications spanning from traditional medicine to culinary uses. Besides its antioxidant and anti-inflammatory properties, immunomodulatory, anticancer, and neuroprotective effects have also been documented. Consequently, *A. argyi* may be regarded as a nutraceutical plant with several health advantages (Song et al., 2019). In several Eastern nations, it is referred to as longevity grass due to its warming properties that alleviate various cold-related ailments (Liu et al., 2017).

Essential oils (EOs) encompass a substantial array of bioactive phytochemicals from *A. argyi*, i.e., silvery wormwood, including alcohols, ethers, terpenes, ketones, and organic acids, among others (Abad et al., 2012). Flavonoids, particularly eupatilin and jaceosidin, are prominent nonvolatile bioactive compounds of *A. argyi*, exhibiting diverse pharmacological properties such as anticancer, antioxidant, anticoagulant, and anti-osteoporosis effects (Han et al., 2017; Liu et al., 2020b; Yoshikawa et al., 1996). Moreover, *A. argyi* contained organic acids, terpenes, polysaccharides, and coumarins (Lv et al., 2018). Currently, plant material is almost the only important source for extracting essential bioactive compounds (Jha and Sit, 2022).

For over a century, supplemental lights have been employed in horticulture to promote plant development and growth (Wallace and Both, 2016). In greenhouse production, supplemental lighting, such as light-emitting diodes (LEDs), is often used to enhance light intensity and tailor light spectra, improving crop yield and quality (Sena et al., 2024). LEDs offer numerous advantages compared to conventional lighting systems, including extended life span, reduced dimensions, diminished thermal radiation, enhanced photosynthetic efficiency, and superior safety performance (Bian et al., 2016; Bula et al., 1991). LEDs do not visibly emit heat but generate thermal energy that must be proficiently degenerate from the system to enhance performance (Li et al., 2024). The advantages of LED lighting are numerous, particularly the creation of a species-specific light formula to enhance plant growth and other beneficial characteristics (Bian et al., 2015; Wei et al., 2019). The advancement in genomics and molecular biology has enabled the in-depth investigation of molecular mechanisms, pathways, and potential bioactive components of various medicinal plants (Alami et al., 2022). Furthermore, plant genomics delivers insights into the biochemical bases and genetics of medicinal properties, facilitating the identification of key enzymes, genes, and secondary metabolites involved in therapeutic effects (Cheng et al., 2021).

This study employed LEDs as a light source to investigate the adaptive responses of *A*. *argyi* physiological parameters, transcriptomics, and metabolomics profile. Moreover, the metabolite results were aligned with the RNA sequencing (RNA-seq) data under the same, i.e., BR vs. RB, BR vs. W and RB vs. W, supplemental light conditions. In summary, secondary metabolite production in plants particularly via lateral branches is highly influenced by environmental factors, specifically light combination in BR then BR to W and RB to W quality, and leads to phytochemical accumulation. Understanding this mechanism will provide a theoretical basis for optimizing light conditions for precision cultivation and improving the production of valuable bioactive compounds in *A. argyi.*

The three supplemental spectra examined in this study—blue:red (BR, 3:1), red:blue (RB, 3:1), and white light (control)—were selected to resolve the relative and combined contributions of the two spectral bands most efficiently perceived by the principal plant photoreceptors, the blue/UV-A-absorbing cryptochromes and phototropins (peak absorption ≈ 450 nm) and the red/far-red-absorbing phytochromes (≈660/730 nm). Fully monochromatic red-only irradiation is well documented to induce a “red-light syndrome” in many species, including impaired stomatal conductance, reduced photosynthetic electron transport, and abnormal leaf morphology, because cryptochrome- and phototropin-dependent pathways remain unstimulated; conversely, sole blue light, while promoting stomatal opening and compact growth, can elevate reactive oxygen species (ROS) accumulation and photo-oxidative stress at high fluence and drives photosynthetic electron transport inefficiently when supplied alone. A reciprocal 3:1 mixed-spectrum design was therefore used against a white-light control, following established practice in LED horticultural lighting research (Bian et al., 2015; Wei et al., 2019; Liu et al., 2020); contrasting the two ratios allows the dominant-color effect to be inferred while avoiding the physiological extremes associated with single-waveband irradiation.

## Materials and methods

### Plant material and growth conditions

The study was conducted from January to February 2025 in the compartment of a Venlo-type glasshouse (8 m × 8 m in size) at the College of Life Sciences, Northwest Agriculture and Forestry University, Yangling, China (52°N, 6°E). Potting soils in a plastic tray were filled and *A. argyi* roots were sown and then covered by vermiculite. Following regular observation, once true leaves have become visible, distinct seedlings were carefully detached and moved to plastic pots with a 19-cm diameter. Plant cultivation took place in measured settings and under stringently regulated conditions as described by Ruamrungsri et al. (2025). Briefly, 68% relative humidity and a temperature between 16 and 18°C were maintained. To mimic the natural light cycle, a 12-h light/12-h dark photoperiod was maintained. Plants were irrigated, with each plant receiving 100 mL three times per week. The composition of the nutrient solution followed exactly that which supports optimal plant growth: phosphorus at 37 mg/L, nitrogen at 143 mg/L, sulfur at 83 mg/L, calcium at 85 mg/L, boron at 0.21 mg/L, iron at 3.86 mg/L, magnesium at 63 mg/L, zinc at 0.02 mg/L, manganese at 0.04 mg/L, potassium at 186 mg/L, molybdenum at 0.05 mg/L, and copper at 0.01 mg/L. LED systems was used to provide supplemental lighting, with treatments varying in both intensity and spectral composition.

### Light treatment conditions and measurements

Light treatments commenced upon shifting the seedlings to the pots and continued for 4 weeks prior to harvesting and measurements of various morphological and physiological traits. The experiments were performed, each comprising three light treatments using LED modules that supplied supplemental white (Res Module, Philips, NL), red (Lumileds, NL), and blue (Res Module, Philips, NL) light for each treatment. Three different LED supplemental lights in two different combinations were tested: blue:red (3:1), red:blue (3:1), and white (control). The detailed light potentials for each LED grouping were maintained as described by Liu et al. (2020a).

### Quantification of growth and physiological traits

Following exposure to LED lights, three plants from each treatment were randomly chosen to evaluate growth and physiological traits until 4 weeks. The plant height (cm), stem diameter (mm), number of leaves and lateral branches (count per plant), shoot fresh weight (g), and shoot dry weight (g) were determined. Keeping in view the physiological traits and growth, 4^th^ week for plant growth hormone, transcriptome, and metabolome analysis.

### Impact of light on plant growth hormone

The hormone-mediated plant responses to supplemental light were measured as described by Brini et al. (2022). Plant hormones (e.g., auxins, gibberellins, abscisic acid, and cytokinin) were extracted from plant tissues and quantification was performed by using high-performance liquid chromatography (HPLC) (Brini et al., 2022).

### RNA isolation, transcriptome sequencing, and analysis

Three shoot samples from each treatment were taken after 4 weeks of exposure to supplemental lights and ground into a fine powder in liquid nitrogen, and total RNA was extracted using a plant RNA extraction kit (TIANGEN Beijing, China). A Qubit Fluorometer and an RNA Assay Kit were used to measure the amount of RNA present, and a Bioanalyzer 2100 (Agilent Technologies, CA, USA) was used to assess the integrity of the RNA. The mRNA molecules were used for complementary DNA (cDNA) synthesis on both the first and second strands. The cDNA fragments were amplified and purified before sequencing and the Illumina Hi-Seq platform was used to sequence the purified amplification products. Raw reads were the designation given to the initial sequencing data. After adaptor sequences, mismatches, and low-quality reads were eliminated, clean reads were generated from the raw reads. The *Brassica_napus*_v4.1 was used as reference genome. At the same time, GC content, sequence duplication level, and the clean data’s Q20 and Q30 were determined. All downstream analysis made use of clean, high-quality data.

Downstream analysis such as differential gene expression was carried out via the DESeq R package (version 1.10.1). The *p*-values were adjusted using the Benjamini–Hochberg method in order to manage the false discovery rate (FDR), while differentially expressed genes (DEGs) were identified based on the criteria |log 2 (fold change) | ≥ 1 and an FDR < 0.05. DEGs were subjected to Gene Ontology (GO) analyses, with all DEGs mapped to their corresponding GO terms in the database (Thomas et al., 2022). We utilized the same method to conduct a BLAST analysis of the DEGs against the Kyoto Encyclopedia of Genes and Genomes (KEGG) database to retrieve significantly associated pathways (Kanehisa and Goto, 2000). The number of genes associated with each term or pathway was calculated, and the hypergeometric test was utilized to evaluate the significantly enriched pathways.

### Quantitative RT-PCR and validation of transcriptomics data

Approximately 0.5 μg of total RNA was used for cDNA synthesis quantitative real-time polymerase chain reaction (qRT-PCR) (Vazyme, Nanjing, China) using HiScript Q RT SuperMix. Following a fivefold dilution of the cDNA reaction mixture, a total reaction system of 10 μL, 1 μL of the diluted mixture was utilized as the template. Overall reaction comprised 0.4 μL of 10 μmol/L primers specific to gene and 5 μL of SYBR^®^ Select Master (2×) ABI (Shanghai, China). qRT-PCR was carried out utilizing an Eppendorf real-time PCR instrument (Hamburg, Germany). For normalization, the actin-2 gene was utilized as the reference gene. Each experiment comprised three biological replicates and three technical replicates.

### Metabolomic profiling and analysis of differential metabolites

Metabolites are essential for understanding organism phenotypes and biological processes. Metabolomics analyzes the pathways and networks. Untargeted metabolomics compares experimental and control groups, detecting metabolites and obtaining quantitative information (Hajnajafi and Iqbal, 2025). The analysis of metabolites was typically divided into three parts: sample preparation and extraction, HPLC and MS conditions (AB), and quantitative analysis.

Plant sample preparation and extraction was carried out as reported by Li et al. (2023). Briefly, using vacuum freeze-drying (Scientz-100F), dry samples were extracted and then milled to powder with a grinder. Plant extract (50 mg) in fine powder was mixed with 1,200 μL of pre-cooled 70% methanol containing internal standards, repeating six times every 30 min for 30 s followed by centrifugation for 3 min at 12,000 rpm. The supernatant filtered with a 0.22-μm membrane was transferred to vials for UPLC-MS/MS analysis.

An LC-MS/MS system was used to analyze each sample following instrument protocols (Agarrwal et al., 2014; Nasr Esfahani et al., 2016; Wang et al., 2018). Data acquisition was achieved using Analyst TF 1.7.1 in IDA mode. TOF MS scan: accumulation time 200 ms, *m*/*z* 50–1250, and dynamic background subtraction enabled. Product ion scan: accumulation time 40 ms, *m*/*z* 50–1250, collision energy ±30 V, and energy spread 15 V. Other settings: UNIT resolution, charge state 1, intensity threshold 100 cps, isotope exclusion within 4 Da, mass tolerance 50 mDa, and maximum 12 candidate ions per cycle.

Among the analytical approaches, unsupervised principal component analysis (PCA) was performed using the prcomp function in R (www.r-project.org) following unit variance scaling. Hierarchical cluster analysis and Pearson correlation coefficients were computed and envisaged as heat maps alone using the R package. Differential metabolite analysis was carried out on the basis of VIP >1, i.e., variable importance in projection and ≥1.0 absolute log2 fold change. VIP values resulting from OPLS-DA were performed using the MetaboAnalystR package. Prior to OPLS-DA, data were log2-transformed and mean-centered. To assess model consistency and prevent overfitting, 200 permutation tests were conducted. Finally, KEGG annotation and enrichment analyses of differential metabolites were performed using the KEGG Compound Database.

## Results and discussion

### Impact of supplemental lighting on growth of *A. argyi*

Medicinal plants have been a natural boon for humanity in combating numerous ailments for millennia. With the globalization of the pursuit for enhanced quality of life, there is a discernible increase in the global demand for functional dietary plants (Kotnis et al., 2004). *A. argyi* is a traditional Chinese herb used for treating various ailments, including fever, hemorrhoids, and diseases associated with fever. It has antioxidant, immunomodulatory, anti-inflammatory, anticancer, and neuroprotective properties, making it a nutraceutical plant with numerous health advantages (Bora and Sharma, 2011). Chinese researchers have identified artemisinin from *A. annua*, leading to lifesaving malaria treatments now widely recommended by the WHO. Thus, *A. annua*, long used for their anti-infective properties, remain important sources for developing new therapies. Traditionally, plant parts such as leaves, bark, roots, and flowers are combined to prepare herbal infusions for treating ailments and promoting wellbeing (El-Saadony et al., 2025).

The results of morphological and various growth parameters determined that quantity and quality of LED light strongly influence plant development, such as with BR light providing optimal conditions for vegetative growth, followed by RB light, while white light supports more balanced but comparatively moderate growth (**Figure 1**). Overall, a similar trend was noted after the 2nd, 3rd, and 4th week. After the 4th week, plants exhibit robust growth with the tallest stems, largest leaves, and the densest foliage under BR light. BR light seems to be the most effective for promoting plant morphology followed by RB and white light. The results indicate that both the intensity and spectral composition of LED light play a critical role in regulating plant growth and morphology.

**Figure 1:**
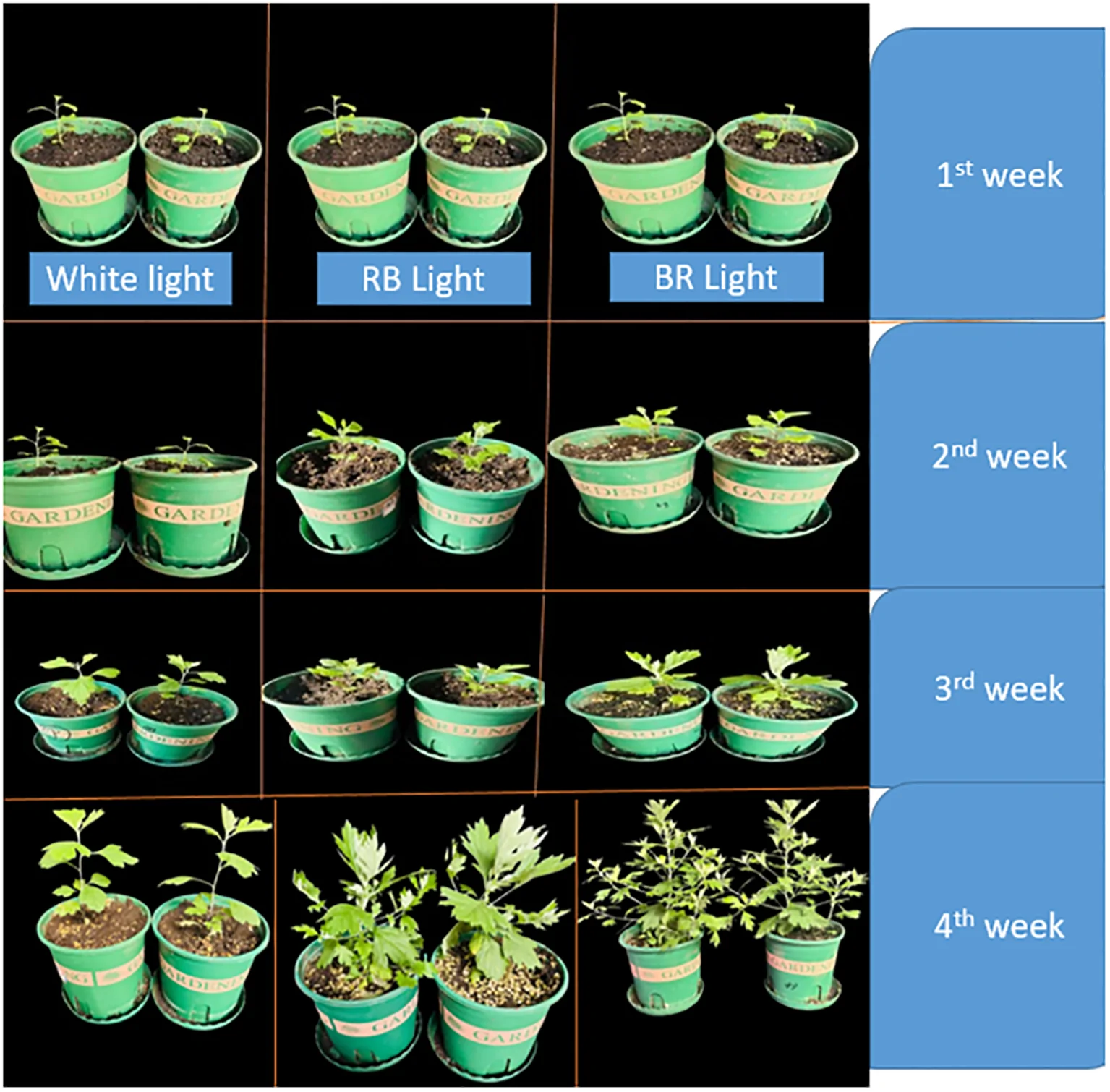
This figure shows the morphological development of *A. argyi* under different supplemental light treatments (BR, RB, and white light) over 4 weeks.

Statistical analysis of plant height indicated a significant difference (*p* ≤ 0.05) across various light ratios and weeks. The application of BR light increased the plant height to 109%, 100%, 78%, and 8.6% after the 1st, 2nd, 3rd, and 4th week (**Figure 2A**). As far as number of leaves is concerned, the increase was 51.61%, 58.33%, 37.20%, and 4.2% after the 1st, 2nd, 3rd, and 4th week, respectively, as compared to RB and white light. A similar trend was noted in stem diameter, shoot fresh weight, shoot dry weight, and lateral branches. These results highlight that the BR (3:1) light ratio is highly effective in promoting plant growth compared to RB and white light (**Figures 2B–F**).

**Figure 2:**
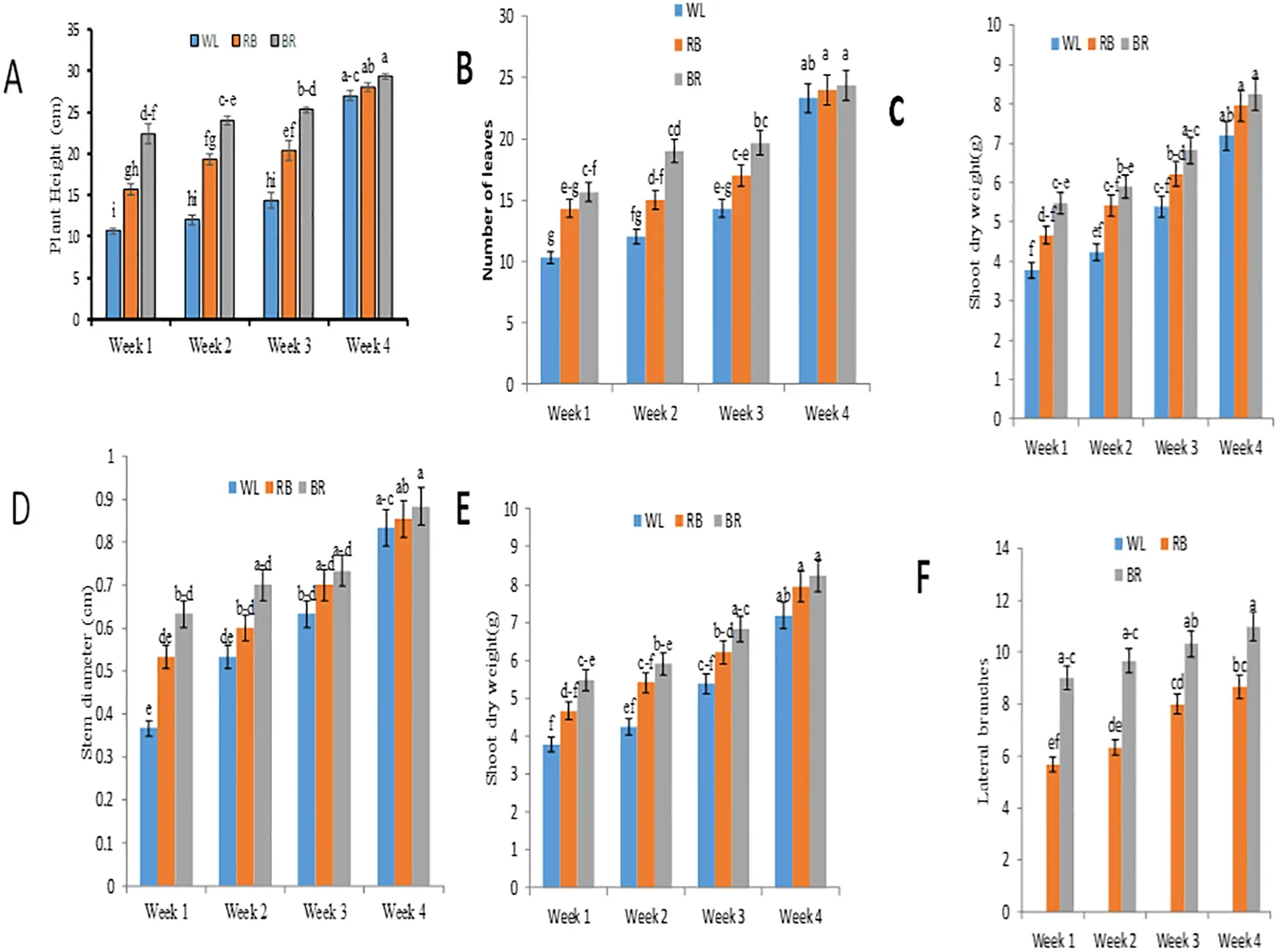
Plant growth metrics under different supplemental light treatments. **(A)** Plant height, **(B)** number of leaves, **(C)** stem diameter, **(D)** shoot fresh weight, **(E)** shoot dry weight, and **(F)** lateral branches. Data were analyzed using one-way ANOVA, and Tukey’s test for multiple comparisons was used to determine statistical significance (*p* ≤ 0.05). Letters above the bars represent groups with significant differences.

In greenhouse production, supplemental lighting, such as LEDs, is often used to enhance light intensity and improve crop yield and quality (Appolloni et al., 2021). This study used LEDs as a light source to investigate the adaptive responses of *A. argyi* under supplementary blue, red, or white light in various combinations. The results of our study corroborated that light quality significantly influences plant development, likely due to variations in photosynthetic efficiency and photomorphogenic responses.

### Effects of supplemental light on plant growth hormones

Plants are generally trained to perceive and respond to wavelengths of light such as red light, blue light, and far-red light (Azizi et al., 2025). The different wavelengths are detected by different photoreceptors, which are made up of a protein covalently bonded to a light-absorbing pigment called a chromophore. BR light enhances levels of IAA and ZR, indicating improved shoot growth and morphological development. BR light promotes moderate levels of most hormones and maximizes ZR, supporting robust morphological development. RB light enhances BR but is less effective in stimulating growth-related hormones like IAA and ZR. White light significantly boosts IAA and ZR, crucial for shoot elongation and cell division, but it shows reduced levels of GA3 and JA-Me, indicating less contribution to elongation and stress responses (**Table 1**).

**Table 1:** Impact of various supplemental lights on plant growth hormones.

Treatment	GA3	ng/g FW	BR	ng/g FW	JA-me	ng/g. FW	IAA	ng/g. FW	ZR	ng/g. FW
BR light	0.81	4.06	0.65	4.56	0.75	18.25	0.51	28.90	0.99	3.00
RB light	0.80	4.13	0.65	4.56	0.76	17.94	0.50	29.60	0.98	3.06
White	0.71	5.08	0.60	5.14	0.45	36.68	0.30	46.62	0.82	4.44

GA3, gibberellic acid; BR, brassinosteroid; JAME, jasmonic acid methyl ester; IAA, indole-3-acetic acid; ZR, zeatin riboside. Values are the mean of three biological replicates per treatment. Plant hormones were quantified by high-performance liquid chromatography (HPLC), and treatment means were compared by one-way ANOVA followed by Tukey’s multiple-comparison test (*p* ≤ 0.05).

The results indicate that different light spectra distinctly modulate endogenous hormone levels. In particular, BR light enhanced the accumulation of IAA and ZR, both of which are key regulators of cell division and shoot development. These hormone-enhanced levels propose that BR light offers promising conditions for improved morphological traits, including enhanced biomass accumulation and shoot elongation (Wang et al., 2025). These findings are consistent with the well-known instances of blue light regulating chlorophyll synthesis, stomatal activity for photosynthetic efficiency, and growth promotion. Interestingly, BR light, while maximizing ZR contents, also maintained moderate levels of most other hormones, representing a balanced hormonal profile that supports robust plant development (Jiang et al., 2025). In contrast, RB light with a 3:1 ratio showed a relatively higher accumulation of brassinosteroids but was less effective in stimulating key growth-promoting hormones such as IAA and ZR. This suggests that although RB light may contribute to certain aspects of plant growth regulation, while it does not optimize the hormonal balance required for maximal shoot development as reported previously (OuYang et al., 2015). These findings highlight how important light quality is for controlling the plant hormone biosynthesis and signaling of plant hormones. Therefore, in controlled environment agriculture, optimizing light spectra particularly through the combination of BR wavelengths can be an effective strategy to manipulate plant growth, raise overall production, and improve morphological features.

### Transcriptome profiling and quality analysis of data

The summary of RNA-seq results shows high-quality transcriptomic data across all (BR, RB, and white) supplemental light treatments. Following quality filtering, the ratio of a large number of reads was retained as total reads in all treatments, showing nominal data loss during preprocessing. The total number of raw reads attained from each treatment of *A. argyi* was between 44,183,368 and 50,746,022, confirming the wide range of transcriptome coverage and representing sufficient sequencing depth for downstream analysis. Moreover, the mapped reads BR vs. RB 42,839,595, BR vs. W 37,102,935, and RB vs. W 33,285,143 show that the reference is compatible for the exposure of supplemental lights to *A. argyi* and that the sequencing data are representative of the *A. argyi* transcriptome. The mapped ratio 83.47% to 87.06% recommends nominal sequencing artifacts, supporting the specificity and quality of the dataset. Similarly, Q30 above 94% and Q20 above 97%, which are high values, signify excellent sequencing accuracy, ensuring confidence in the generated data (**Table 2**).

**Table 2:** Summary of *A. argae* cDNA samples sequenced using Illumina genome analysis.

Treatment name	BR vs. RB	RB vs. W	BR vs. W
Raw reads	50,746,022	38,633,010	44,183,368
Total reads	50,219,702	38,242,412	43,729,641
Mapped reads	42,839,595	33,285,143	37,102,935
Unique reads	28,349,756	24,594,610	28,414,638
Mapped ratio	85.37	87.06%	83.47%
Q20	97.98%	97.96%	98.03%
Q30	94.17%	94.13%	94.32%
GC	45.23%	44.41%	43.42%

In short, sequencing data show sequencing results for *A. argyi* samples with high quality and indicate that the sequencing process was reliable and effective. The sequencing summary obtained in the present study reveals high-quality transcriptomic data across BR, RB, and white supplemental light conditions, which is consistent with established standards in RNA-seq studies (Wang et al., 2009; Zhang et al., 2010). Previous studies have reported that a sequencing depth of 20–30 million reads is generally adequate for reliable gene expression profiling, while higher depths further improve sensitivity and detection of low-abundance transcripts (Antoszewski et al., 2026).

Furthermore, all data that were used in this study were potentially reproducible in terms of correlation for each condition between three biological replicates (*R* = 0.95–0.98, *p* < 0.001) (**Figure 3A**). For each RNA sample, heat map of coverage identified numerous regions with transcript richness that was uniformly low or uniformly high (**Figure 3B**). Additionally, the box plots showed that the positions of the distributions of the gene expression values in all three samples of *A. argyi* are usually similar, although there is substantial variance in the dissemination of the expression level (log_2_RPKM) of expressed annotated genes between three biological replicates of each sample (**Figure 3C**). In alignment with hierarchical clustering, a PCA plot on the basis of three gene expression profiles resulted in a pure separation between the light conditions and three biological replicates (**Figure 3D**). The consistency of the sequencing data and strong correlation ensures that the observed differences are biologically meaningful rather than artifacts (Yu et al., 2024).

**Figure 3:**
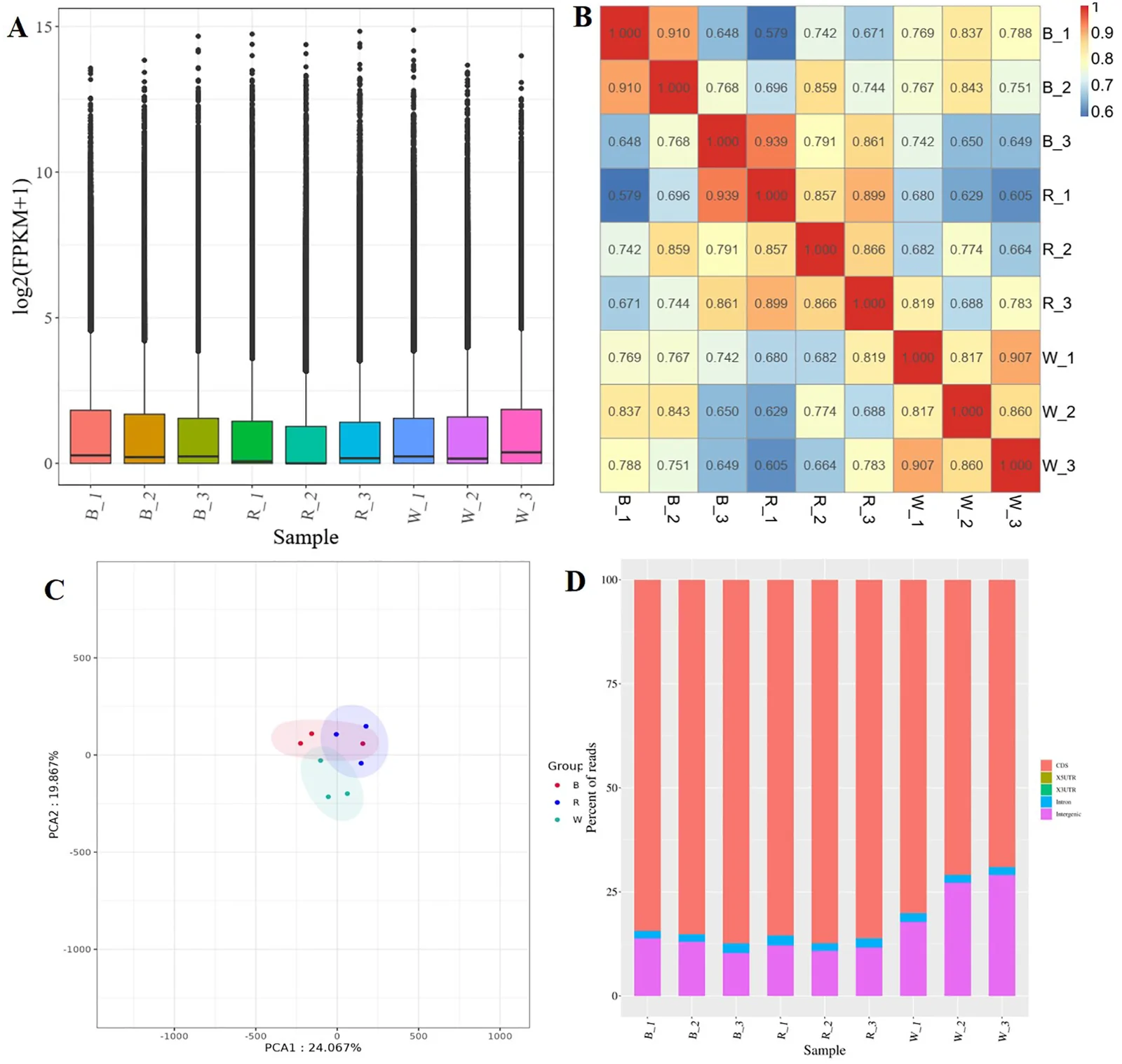
Statistical quality analysis of RNA sequencing data. **(A)** Correlation analysis (*R* = 0.95–0.98, *p* < 0.001). **(B)** Heat map of coverage, i.e., uniform distribution of expression levels across samples. **(C)** Principal components analysis, separation of samples in the PCA plot. **(D)** Box plot analysis shows the distribution of gene expression values across the different conditions.

### Assessment of gene expression profiles and sample clustering

The distribution of gene expression levels in all samples (B, R, and W groups) is similar, according to the box plot of log2(FPKM + 1) values where spread and comparable median expression show no significant technical bias and that normalization was successful. This reliability suggests that the datasets are reliable and suitable for downstream comparative analysis. In sample correlation analysis, replicates show high correlation coefficients (generally >0.85), particularly within the same light treatments (e.g., B_1–B_3, R_1–R_3, and W_1–W_3) (**Figures 4A–C**). In contrast, correlations between different treatment groups are relatively lower, indicating distinct transcriptional responses under white, red, and blue light.

**Figure 4:**
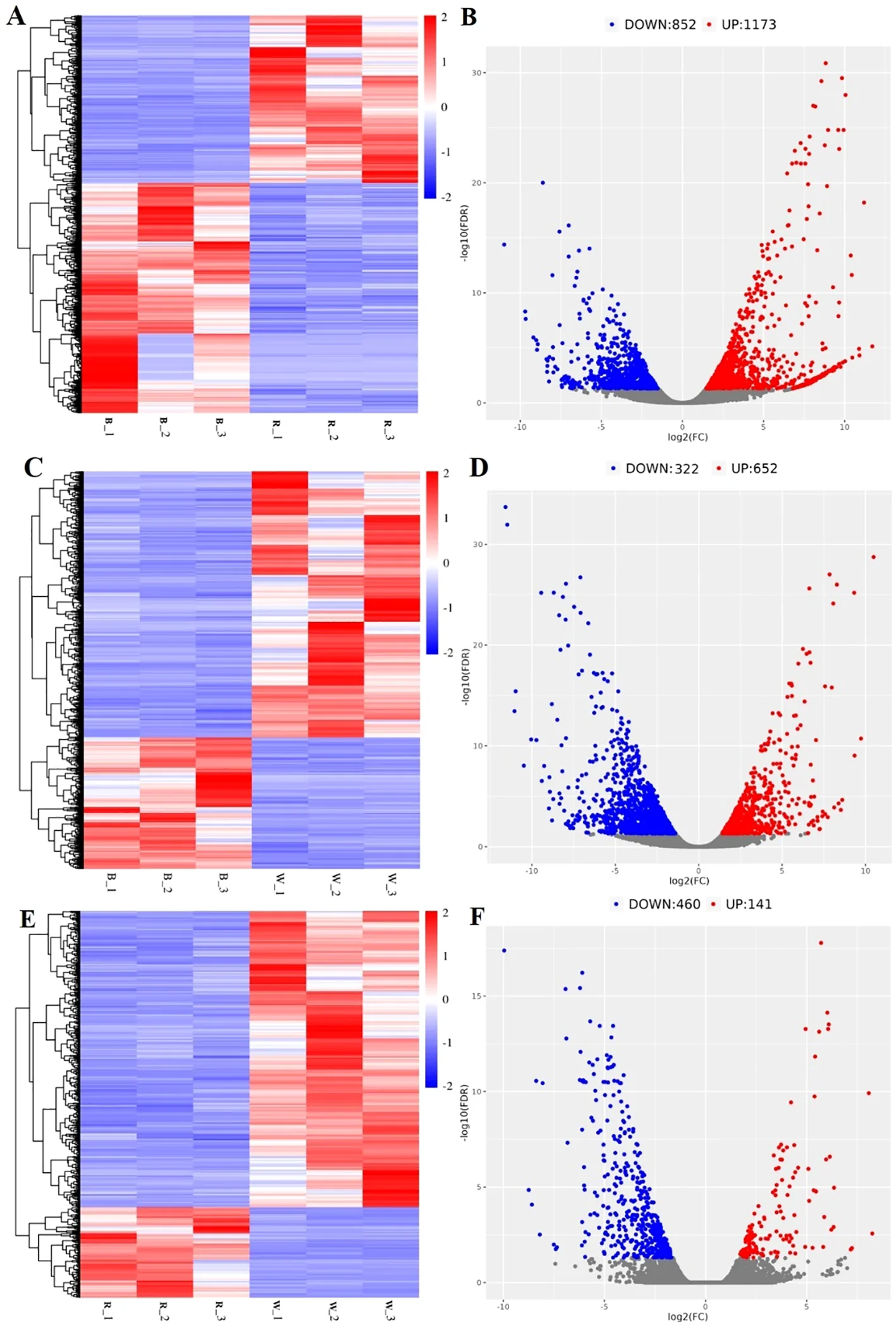
Differentially expressed genes on exposure to supplemental lights. **(A)** Heat map and volcano plot showing the expression profiles of DEGs, i.e., BR vs. RB. **(B)** Heat map and volcano plot showing the expression profiles of DEGs, i.e., BR vs. W comparison. **(C)** Heat map and volcano plot showing the expression profiles of DEGs, i.e., RB and W light. Statistical significance was determined using DESeq analysis with a false discovery rate (FDR) < 0.05 and |log2(FC)| ≥ 1. **(D)** Volcano plot showing differentially expressed genes (DEGs) in CW vs. W comparison (DOWN: 322, UP: 652). **(E)** Heat map showing expression profiles of DEGs in RB vs. W comparison. **(F)** Volcano plot showing DEGs in RB vs. W comparison (DOWN: 460, UP: 141). Statistical significance was determined using DESeq2 analysis with a false discovery rate (FDR) < 0.05 and |log2FoldChange| ≥ 1. Blue dots represent down-regulated genes, red dots represent up-regulated genes, and grey dots represent non-significant genes.

To gain insight into the genetic physiology of *A. argyi* primarily due to the impact of the supplemental light, messenger RNA-seq of the leaf samples following 4 weeks of exposure was carried out. Variation in gene expression was studied to analyze the genes that may participate in light-induced morphological to genetic alteration. Through pairwise comparison such as BR vs. RB, a total of 1,173 upregulated genes and 852 downregulated genes were identified (**Figures 4A, B**); in BR vs. white, 652 upregulated and 322 downregulated DEGs were identified (**Figures 4C, D**), while in RB vs. white, 141 upregulated and 460 downregulated genes were identified (**Figures 4E, F**). The mRNA sequencing results provide a detailed overview of how different light conditions affect gene expression in *A. argyi* leaves after 4 weeks of supplemental light exposure. Moreover, the increasing number of DEGs from BR to RB to W implies that the spectrum of light plays a crucial role in modulating gene expression. This aligns with the notion that various wavelengths distinctly impact morphological and physiological traits (Han et al., 2026). The higher proportion of upregulated genes under BR compared to RB, RB compared to white, and BR compared to white suggests that blue light in combination with red light might activate more genes involved in growth, development, or photosynthesis-related pathways. Emerging evidence demonstrates significant variation in BR responses under various growth conditions between model and crop species. Flowering involves multiple photoreceptors and complex signaling pathways, leading to diverse outcomes under controlled conditions (Kong and Zheng, 2025). Therefore, optimizing BL for lateral branch regulation could be potential findings for further mechanistic studies, especially in horticultural crops, are essential to fully exploit BL in crop production.

### GO category analysis of light-responsive genes

We classified the DEGs’ functions using GO assignments. Three primary GO categories, namely, “biological process (BP)”, “cellular component (CC)”, and “molecular function (MF)”, were used to annotate the enriched genes in the three comparisons.

GO enrichment analysis showed that DEGs across all comparisons mostly associated to core biological processes like cellular processes, metabolism, stimulus responses, and regulation. The BR vs. W comparison had the strongest enrichment (**Figure 5B**), followed by RB vs. W (**Figure 5C**), while BR vs. RB showed weaker enrichment, pointing to higher similarity between BR and RB (**Figure 5A**; **Supplementary Table S1**). Patently, BR vs. W and RB vs. W raised out with enrichment in stress- and defense-related pathways, such as stimulus responses, detoxification, and immune processes, hinting at bolstered stress adaptation in those groups. Cellular component analysis identified membranes and organelles as primary locations, whereas molecular function analysis underlined binding and catalytic activity along with transporter, transcription regulator, and antioxidant roles. In short, stress-responsive and metabolic pathways drive the key differences.

**Figure 5:**
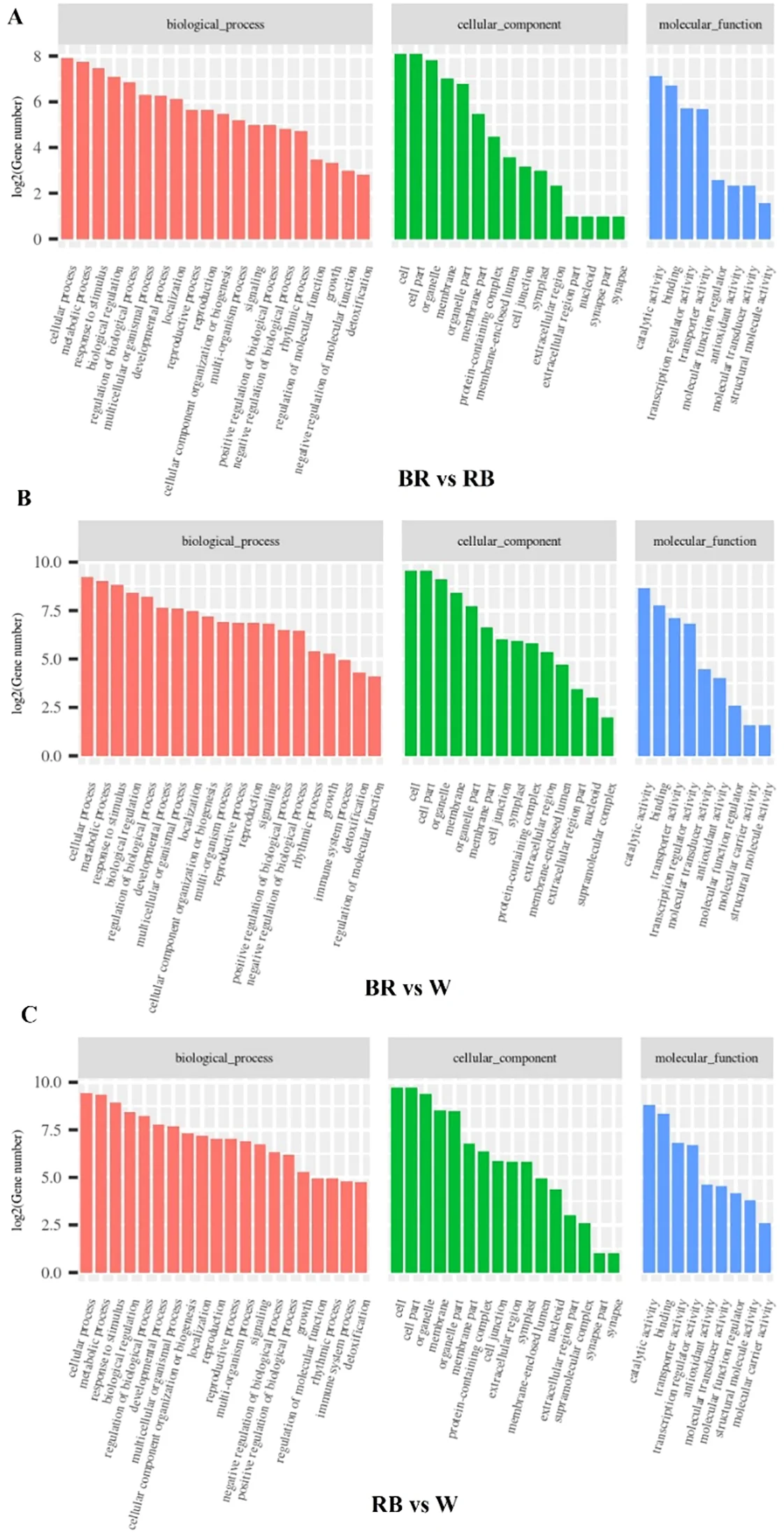
GO term enrichment analysis of the top 20 DEGs. **(A)** Comparison of BR vs. RB. **(B)** Comparison of BR vs. (W) **(C)** Comparison of RB vs. (W) The top 20 enriched GO terms for biological processes, cellular components, and molecular functions are displayed. Statistical significance was determined by Fisher’s exact test, with a *p*-value threshold of <0.05.

Additionally, GO analyses showed that the DEGs induced by supplemental blue light impact enriched the GO terms linked to regulation of auxin biosynthetic process, circadian rhythm, and regulation of shoot development, which leads to the well-developed shoots and lateral branches in *A. argyi*.

The findings support earlier research showing that blue light promoted the growth of the length of lateral branches, which significantly decreased leaf area (Si et al., 2022); however, light energy utilization and its rate were equal among all conditions, contrary to Si et al. (2022). Si et al. reported that a higher amount of blue light is needed compared to other light treatments (Wang et al., 2016). This suggests that the utilization of blue light in *A. argyi* farming is both cost-effective and practical, offering a dual benefit of promoting desirable plant growth characteristics while maintaining efficient energy utilization.

### Significant pathway enrichment analysis of DEGs

KEGG analysis revealed that differences between blue and red light primarily affected photosystem function and circadian rhythm, and when compared with white LED light, carbon metabolism (sugars), secondary metabolites (defense compounds), and stress signaling pathways (MAPK and hormones) were highly enriched. The DEGs associated with these pathways are detailed in **Supplementary Table S2**. In the BR vs. RB comparison (**Figure 6A**), pathways such as circadian rhythm plant; aspartate, alanine, and glutamate metabolism pathway; leucine, isoleucine, and valine degradation pathway; arginine and proline metabolism; porphyrin metabolism; terpenoid backbone biosynthesis; and ABC transporters. In the circadian rhythm plant pathway, the upregulated genes were Aarg03G020500, Aarg15G010000, Aarg07G011210, Aarg07G024120, and Aarg15G023360. The genes Timing of CAB expression 1 and CheY-like phosphoacceptor are encoded by the two-component response regulator-like APRR1 and FKF1 (**Supplementary Figure S1**).

**Figure 6:**
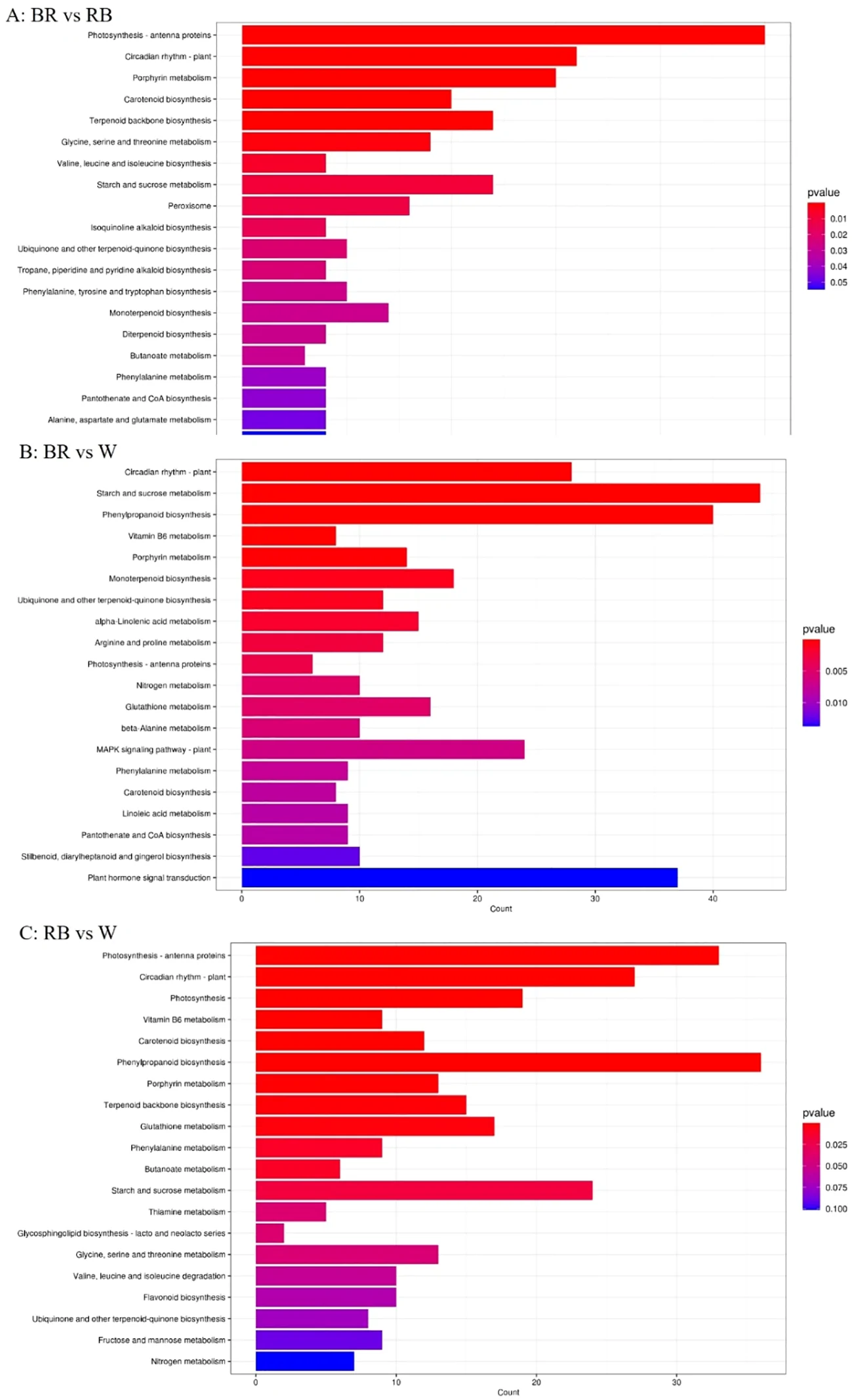
KEGG pathway enrichment analysis of DEGs. **(A)** BR vs. RB, **(B)** BR vs. W, and **(C)** RB vs. (W) This figure illustrates the top enriched pathways identified in the KEGG analysis of DEGs across different light treatments. Pathway enrichment was determined using the hypergeometric test (*p* ≤ 0.05), and the results are presented for each light treatment comparison.

In alanine, aspartate, and glutamine metabolism, Aarg12G003250, Aarg11G011100, Aarg03G008980, and Aarg05G031480 genes were upregulated, which enables asparaginase activity and beta-aspartyl-peptidase activity (**Supplementary Figure S2**). In arginine and proline metabolism, genes Aarg15G020000 and Aarg07G021120 were associated. These genes are known to play a key role in elevating the expression of the ProDH gene in seeds and pollen grains (**Supplementary Figure S3**). In porphyrin metabolism (**Supplementary Figure S4**), Aarg02G028690/Aarg10G059200 genes associated with non-yellow coloring 1 (nyc1)-like (NOL) were upregulated. The chlorophyll b reductase NOL (NYC1-like) oversees catalyzing the degradation of chlorophyll b and maintaining the stability of the photosystem as the degradation of chlorophyll is of great significance to plant growth. Terpenoid backbone biosynthesis Aarg06G004000 and Aarg14G004320 genes were associated, which are associated with the isoprene, an antioxidant to guard plants against stress (**Supplementary Figure S5**). Moreover, the details of each gene associated with different pathways are given in **Supplementary Table S2**.

The study used KEGG analyses to identify pathways highly enriched by DEGs, including circadian rhythm plant; alanine, aspartate, and glutamate metabolism; valine, leucine, and isoleucine degradation pathway; arginine and proline metabolism; porphyrin metabolism; terpenoid backbone biosynthesis; and ABC transporters. The most important genes and associated pathways were genes Timing of CAB expression 1 and CheY-like phosphoacceptor, which are encoded by the two-component response regulator-like APRR1, proline dehydrogenase (ProDH), and chlorophyll b reductase NOL (NYC1-like) catalyzing the degradation of chlorophyll b and maintaining the stability of the photosystem, isoprene, an antioxidant to guard plants against stress. As described previously, plants may prioritize resource allocation to apical growth over lateral branching if CAB1 expression is misaligned with environmental light conditions (Schneider et al., 2019).

Significantly, the results suggest that misalignment of light-responsive genes such as CAB1 can change resource allocation toward apical growth at the expense of lateral branching. This offers possible targets for maximizing agricultural yield and stress resilience as well as mechanistic insight into how environmental cues control plant design.

### Validation and expression analysis of genes encoding key enzymes

Using actin as the reference gene, nine DEGs associated with porphyrin metabolism, arginine and proline metabolism, and terpenoid backbone biosynthesis were validated by qRT-PCR. All primers used are listed in **Table 3**. The results demonstrated that the expression trends observed in RNA-seq were consistent with those obtained from qRT-PCR, confirming the reliability of the RNA-seq data (**Figure 7**).

**Figure 7:**
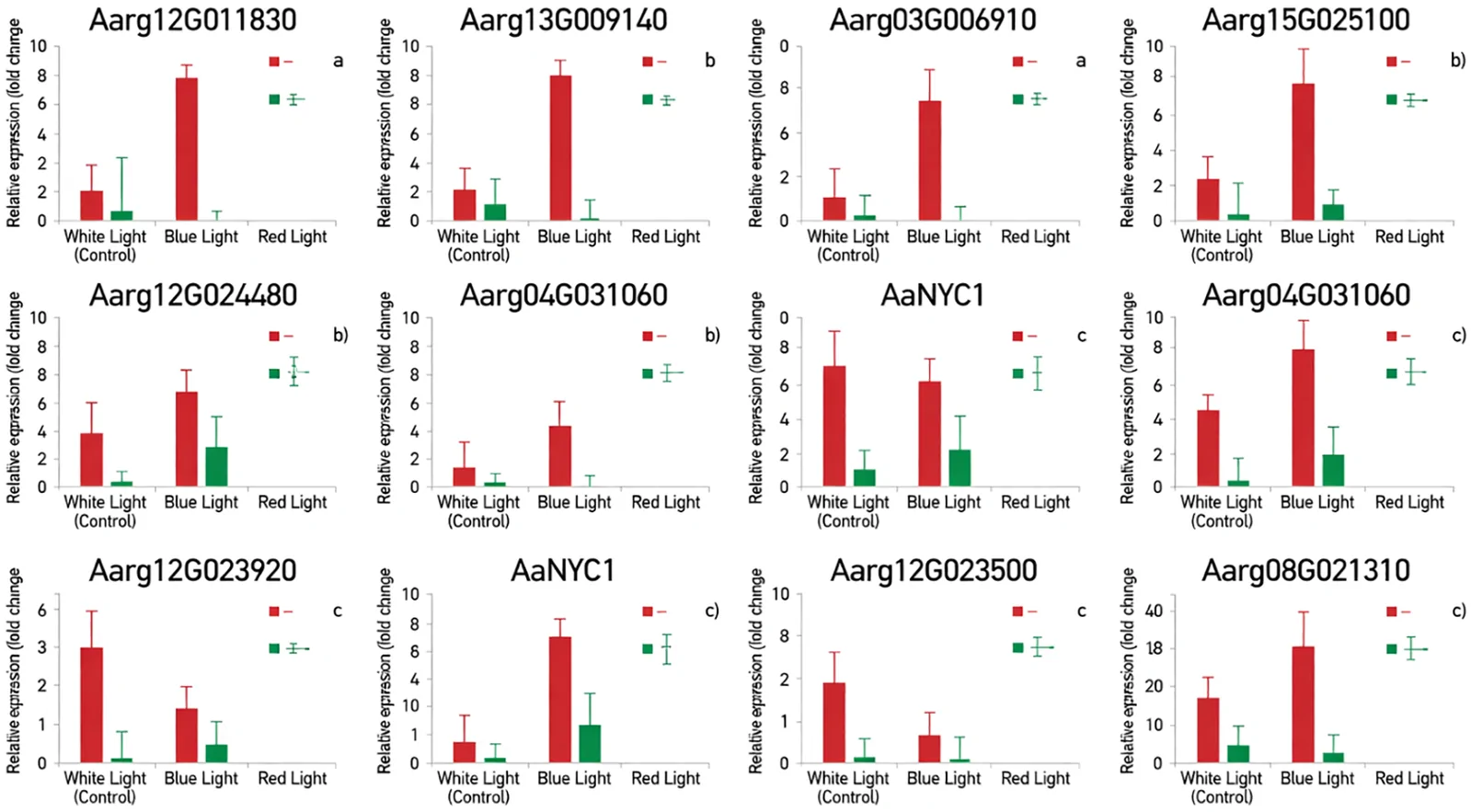
qRT-PCR validation of differentially expressed genes in Artemisia argyi lateral branches under blue and red light. **(A)** Aarg12G011830, **(B)** Aarg13G009140, **(C)** Aarg03G006910, **(D)** Aarg15G025100, **(E)** Aarg12G024480, **(F)** Aarg04G031060, **(G)** AaNYC1, **(H)** Aarg04G031060, **(I)** Aarg12G023920, **(J)** AaNYC1, **(K)** Aarg12G023500, **(L)** Aarg08G021310. Different lowercase letters (a, b, c, d) above the bars indicate statistically significant differences (p < 0.05) as determined by Duncan's multiple range test. Red: Upregulated, Green: Downregulated. Data are presented as mean ± SD (n=3).

**Table 3:** Primers used for RT-qPCR analysis.

Gene ID	Forward Primer (5′→3′)	Reverse Primer (5′→3′)	Amplicon Size (bp)
***Aarg12G011830***	CTCCACCGAAGTCTCCTCC	GGTGTTTTTGGTGGATCGAT	156
***Aarg13G009140***	GATGCAGATCTTCGTGAAAAC	CAGCCAAAGCCATCAAGAAG	142
***Aarg03G006910***	GGTGTTAGGAAGAGGTGCAA	CCCGACAAACCTTTCAATTC	168
***Aarg15G025100***	CTTCCACAAAGCCGATGAAA	TCCACGAAGAAGACCTCCTT	175
***Aarg12G024480***	GGGTCTAGATTAGAGGACACTATT	CAGCACATTCCATCTGAATACG	149
***Aarg04G031060***	GGAGAATGAAAGGTTCTTTTTTAGC	CTTGTGCTAAAAAAGAACCTTCATTC	162
***Aarg12G023920***	GAAATGCCACTGTCAACTCG	CACAAGAATTGAGCTCTAACACG	158
***Aarg12G023500***	GCTGTTCCTTGCTGATGCTA	CAAACACCTTCATCAGCCTTC	153
***Aarg08G021310***	GATATGAGCTGTTTGACCATAGTC	CGTGCTTGCCAAGTCCTTC	167
***AaNYC1***	CCCTCGGATGAGAGTTGTAAAG	CAAACCAACGTCCTCGTCTTA	110
***Actin* (reference gene)**	TACTGCCCTGGCTCCTAGCA	TGGACAGTGAGGCCAGGATA G 54	142

### Differential metabolite profiling of *A. argyi* under supplemental light treatments

To gain deeper insight into the metabolic changes in *A. argyi* in lateral branches and integrations of transcriptomics and metabolomics, GC-MS analysis was performed to profile the primary metabolites. A total of 3,563 metabolites were identified. Among them, 945 metabolites were identified as differentially significant under BR vs. RB light impact, of which 459 were downregulated and 486 were upregulated. Under BR vs. W impact, 907 metabolites were identified as differentially significant, of which 440 were downregulated and 467 were upregulated. Under RB vs. W light impact, 1,036 metabolites were identified in total, with 579 downregulated and 363 upregulated (**Table 4**). The samples (including QC samples) were analyzed by PCA to get an initial idea of the overall metabolite differences between groups of samples and the magnitude of variability between samples within groups. The PCA results showed a trend of metabolite separation between groups such as with PC1 (37.86%) distinguishing white light from blue and red light, and PC2 (15.95%) separating blue and red light treatments. Replicates within each group clustered tightly, representing high reproducibility (**Figure 8**).

**Table 4:** Summary of differential metabolite under various light impacts.

Group name	All sig diff	Downregulated	Upregulated
BR vs. RB	945	459	486
BR vs. W	907	440	467
RB vs. W	943	579	363

**Figure 8:**
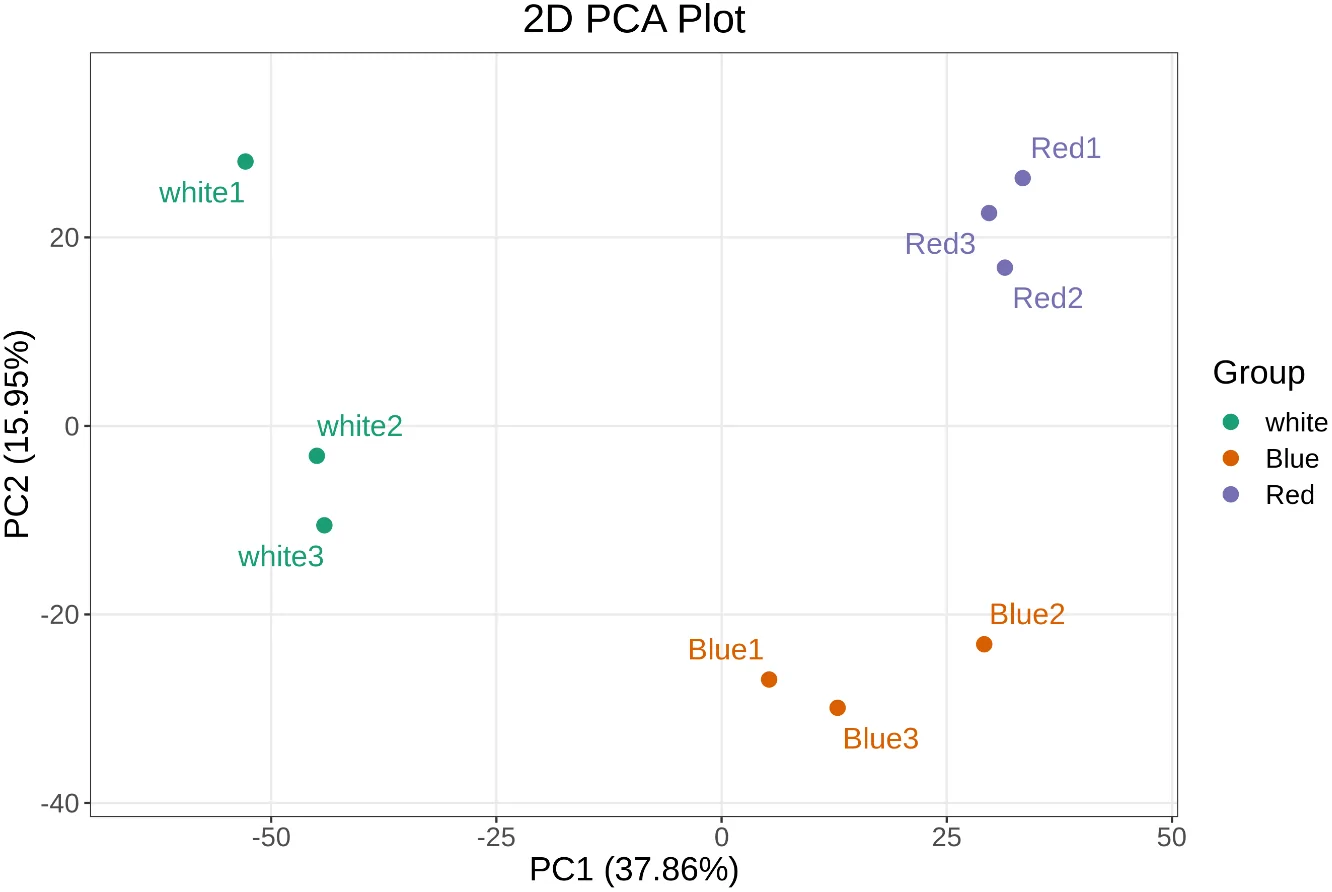
2D principal component analysis plot summarizes the overall variation in metabolite profiles of *A. argyi* under different light treatments.

These results demonstrate that light quality significantly influences the metabolic profiles of *A. argyi*. Moreover, GC-MS identified amino acids and derivatives, e.g., serine, alanine, and aspartic acid, and organic acids (e.g., glyceric acid, citric acid, and malic acid). Unbiased PCA showed that the metabolites of samples treated with various combinations of red and white light were different from those of blue light.

Moreover, by OPLS-DA and one-way analysis of variance (ANOVA), a total of 174, 129, and 81 differential metabolites were identified in BR vs. RB, BR vs. W, and RB vs. W, respectively (**Figure 9**).

**Figure 9:**
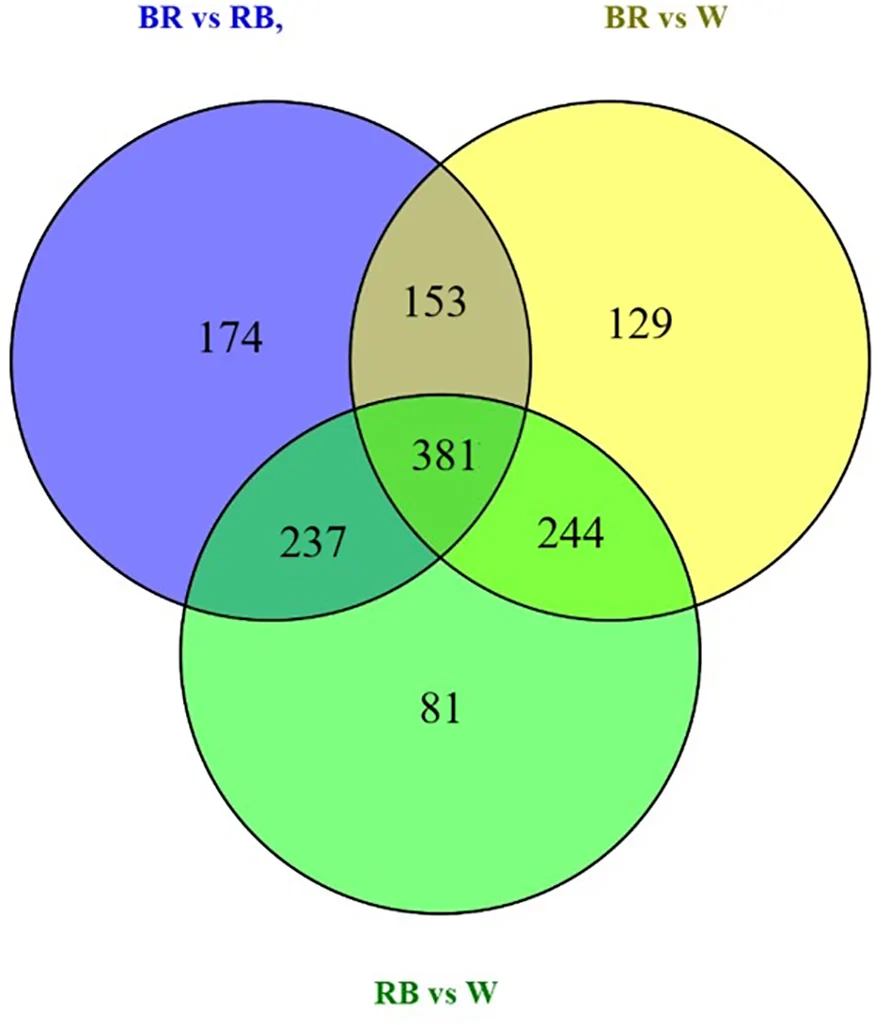
Venn diagram showing the overlap of differentially accumulated metabolites among three comparisons (BR vs. RB, BR vs. W, and RB vs. W) in *A. argyi*.

The results demonstrate that the blue–red light combination markedly enhances metabolite accumulation in *A. argyi*, as evidenced by the upregulation of 486 metabolites including bracteatin, neoabietic acid, cannabigerol, methyl arachidonate, and cephalomannine compared to red–blue light treatments. Conversely, 459 metabolites, such as catalposide, perillaldehyde, and myristicin, were downregulated under the same conditions (**Supplementary Table S3**). Notably, the combined red–blue light treatment also exhibited a stimulatory effect on metabolite content following blue–red light. KEGG pathway enrichment analysis indicated that blue light significantly modulated a wide range of metabolic pathways, particularly those associated with amino acid (e.g., glutamate, arginine, and tryptophan), carbohydrate (e.g., glycolysis and TCA cycle), lipid (e.g., glycerolipid), and secondary metabolite (e.g., alkaloids, flavonoids, and terpenoids) biosynthesis. In addition, pathways related to genetic information processing (e.g., aminoacyl-tRNA biosynthesis) and environmental signaling (e.g., ABC transporters, phosphatidylinositol signaling, and plant hormone signal transduction) were also affected, suggesting that blue light not only drives metabolic reprogramming but also enhances adaptive responses (**Figure 10**). Overall, the dominance of metabolic pathways under blue light highlights its role in promoting both primary and secondary metabolite biosynthesis in *A. argyi*, potentially contributing to its medicinal properties.

**Figure 10:**
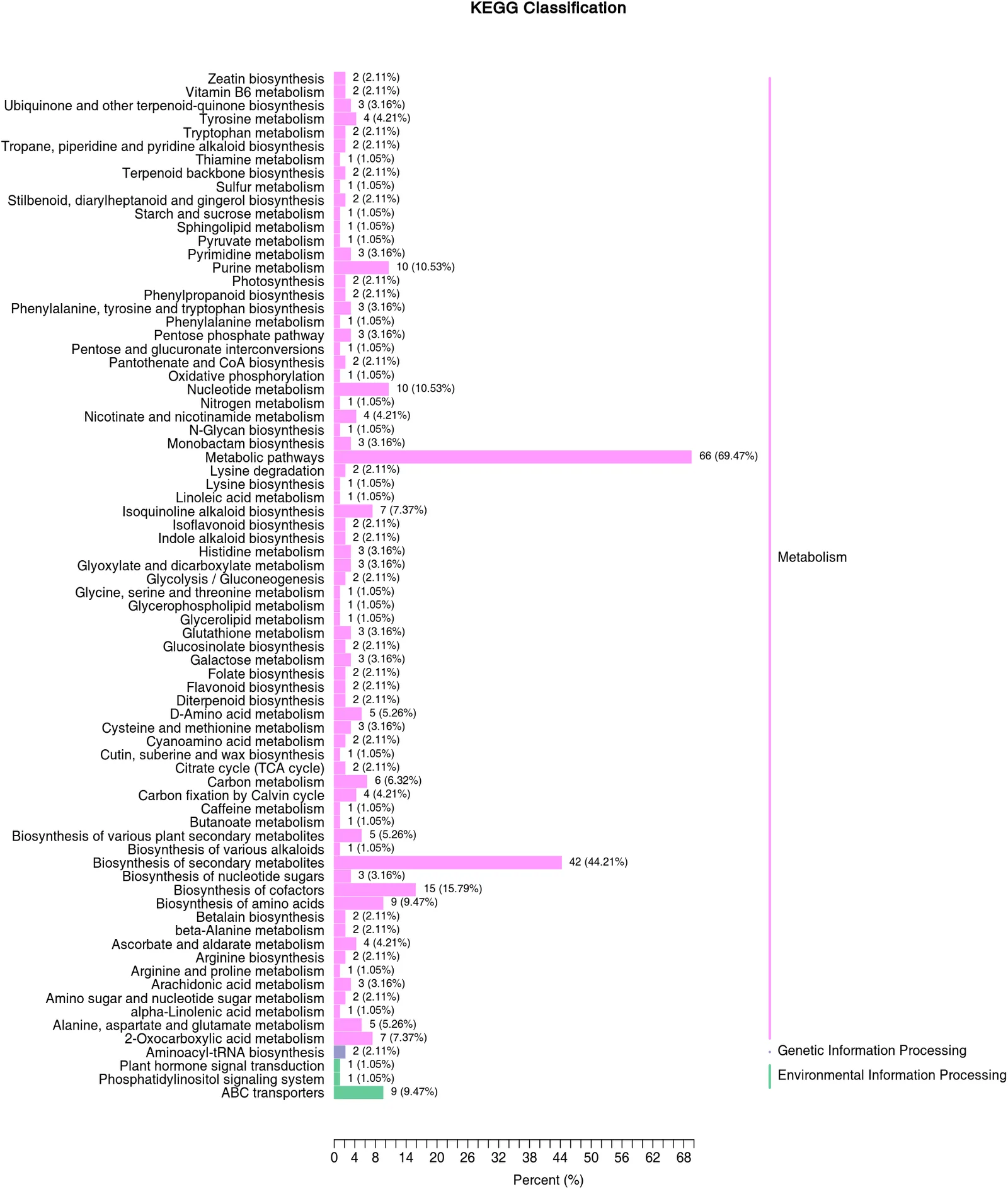
KEGG pathway classification of differentially accumulated metabolites by blue light treatment in *A. argyi*. Data were analyzed using KEGG pathway enrichment analysis, with a *p*-value threshold of <0.05.

However, red vs. white promoted a broad reprogramming of metabolic activity in *A. argyi*, particularly boosting secondary metabolite biosynthesis and amino acid metabolism, likely contributing to enhanced defense and medicinal compound production. The enrichment of nucleotide and cofactor biosynthesis also suggests increased cellular biosynthetic demand. While metabolism dominated the changes, involvement of transporter and signaling pathways implies red light also triggers adaptive physiological responses (**Figure 11**).

**Figure 11:**
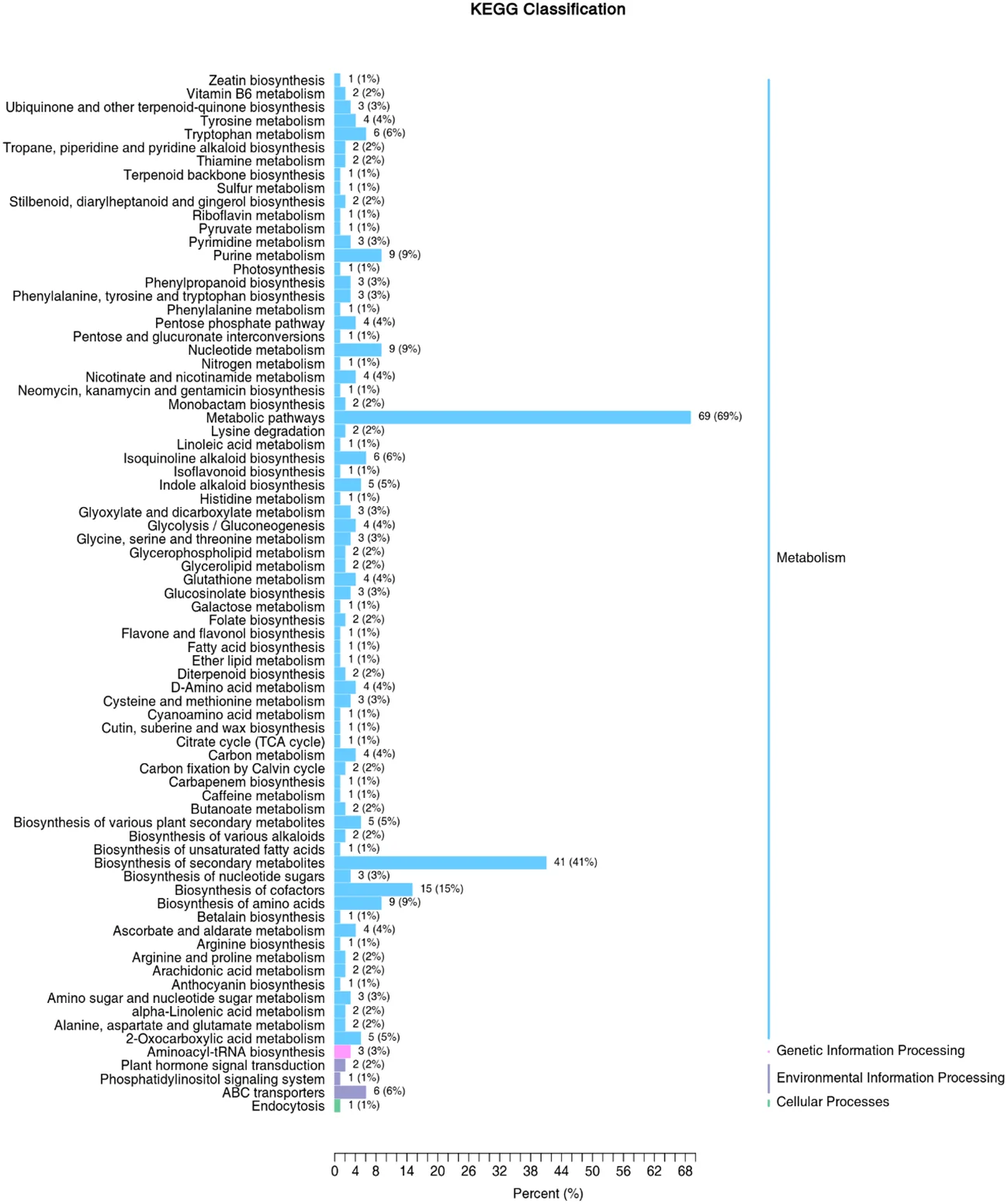
KEGG pathway classification of differentially accumulated metabolites in *Artemisia argyi* under red light compared to white light.

Although the present design compared mixed blue:red (BR, 3:1) and red:blue (RB, 3:1) supplemental spectra against a white-light control rather than fully monochromatic red- or blue-only regimes, the contrasting KEGG enrichment patterns obtained for the BR- and RB-dominant treatments are broadly consistent with reports that examined sole red and sole blue irradiation in other species. Sole red light typically triggers what has been termed “red-light syndrome”, comprising reduced stomatal conductance, impaired photosynthetic electron transport, and altered leaf anatomy because cryptochrome- and phototropin-dependent pathways remain unstimulated, whereas sole blue light promotes compact growth and stomatal opening but can elevate ROS accumulation and photo-oxidative stress at high fluence. The BR vs. RB contrast recorded here reproduces this pattern at the pathway level: red-enriched (RB) input over-represented stress-, transporter-, and defense-related signatures typical of a red-light-syndrome-like response, while blue-enriched (BR) input more strongly activated antioxidant enzymes and ROS-associated pathways together with terpenoid and flavonoid biosynthesis, consistent with a blue-light photo-oxidative signature that here translates into enhanced bioactive-compound accumulation rather than damage. This concordance supports the interpretation that the BR/RB reciprocal design captures the same underlying red- versus blue-photoreceptor contributions that monochromatic treatments would reveal directly; nonetheless, we regard side-by-side monochromatic red- and blue-only treatments as a valuable confirmatory experiment and identify it as a priority for follow-up work (see Conclusions).

The study used KEGG analyses to identify pathways highly enriched by DEGs, including circadian rhythm plant; alanine, aspartate, and glutamate metabolism; valine, leucine, and isoleucine degradation pathway; arginine and proline metabolism; porphyrin metabolism; terpenoid backbone biosynthesis; and ABC transporters. The most important genes and associated pathways were genes Timing of CAB expression 1 and CheY-like phosphoacceptor, which are encoded by the two-component response regulator-like APRR1 (Kong and Zheng, 2025), ProDH, and chlorophyll b reductase NOL (NYC1-like) catalyzing the degradation of chlorophyll b and maintaining the stability of the photosystem, isoprene, an antioxidant to guard plants against stress (Bi et al., 2021; Guan et al., 2022). As described previously, plants may prioritize resource allocation to apical growth over lateral branching if CAB1 expression is misaligned with environmental light conditions (Faseela et al., 2020; Schneider et al., 2019).

The metabolite profiling carried out by GC-MS of *A. argyi* under three different light exposures revealed a diverse array of metabolites, including organic acids (e.g., glyceric acid, citric acid, and malic acid) and amino acids and their derivatives (e.g., serine, alanine, and aspartic acid). Metabolite PCA revealed distinct separation between plants exposed to BR and those exposed to RB or white light, representing significant metabolomic shifts, which is in alignment with previous studies that show that light, particularly blue LED, has a significant impact on plant metabolites (Obata and Fernie, 2012). Blue light had the most pronounced effect on metabolite accumulation, as noted by the upregulation of 486 compounds, including bioactive molecules such as bracteatin, methyl arachidonate, cannabigerol, neoabietic acid, and cephalomannine. It proposes a light-dependent regulation of biochemical pathways, highlighting its importance in metabolic engineering and photobiology (Borbély et al., 2022; Wang et al., 2024).

KEGG pathway enrichment analysis of identified metabolites revealed that blue light significantly modulates key metabolic pathways, with significant enrichment in carbohydrate metabolism (e.g., glycolysis and TCA cycle), lipid metabolism (e.g., glycerolipid metabolism), amino acid metabolism (e.g., glutamate, arginine, and tryptophan), and secondary metabolite biosynthesis pathways (e.g., alkaloids, flavonoids, and terpenoids). It reveals that blue light not only drives biosynthesis but also improves the plants and its adaptive physiological capacity (Reichel et al., 2022; Senger, 2008). However, red light compared to white light also induced extensive metabolic reprogramming, such as the upregulation of pathways related to amino acid biosynthesis and secondary metabolites potentially contributing to improved stress tolerance and medicinal value. Enrichment of cofactor biosynthesis and nucleotides represents enhanced biosynthetic demands under red light (Khan et al., 2026).

Furthermore, these findings demonstrate the overlapping and exclusive roles of blue and red light in modulating metabolite profiles in *A. argyi*. It provides persuasive evidence for the optimized use of various light spectra in various combination to enhance the biosynthesis of bioactive compounds, offering valuable guidance for controlled cultivation of *A. argyi* and other medicinal plants. Remarkably, these pathways were consistently enriched in both transcriptome profiling and metabolomic profiling of *A. argyi* under various light spectra, indicating strong concordance between metabolite accumulation and gene expression profiles under blue light treatment.

To integrate these observations, we propose a consolidated signaling model that links the light stimulus to the growth, branching, and metabolic responses recorded in *A. argyi*. The molecular players identified in this study, the two-component response regulator-like APRR1/TOC1, FKF1, ProDH, the chlorophyll b reductase NOL/NYC1, and the terpenoid-backbone genes, are organized into a single proposed protein–protein interaction and directional signaling model (**Figure 12**) that summarizes how blue-enriched (BR) light coordinates photoreceptor perception, the COP1–HY5 hub and circadian clock, hormone signaling and the BRC1/TB1 branching switch, and downstream secondary metabolism.

**Figure 12:**
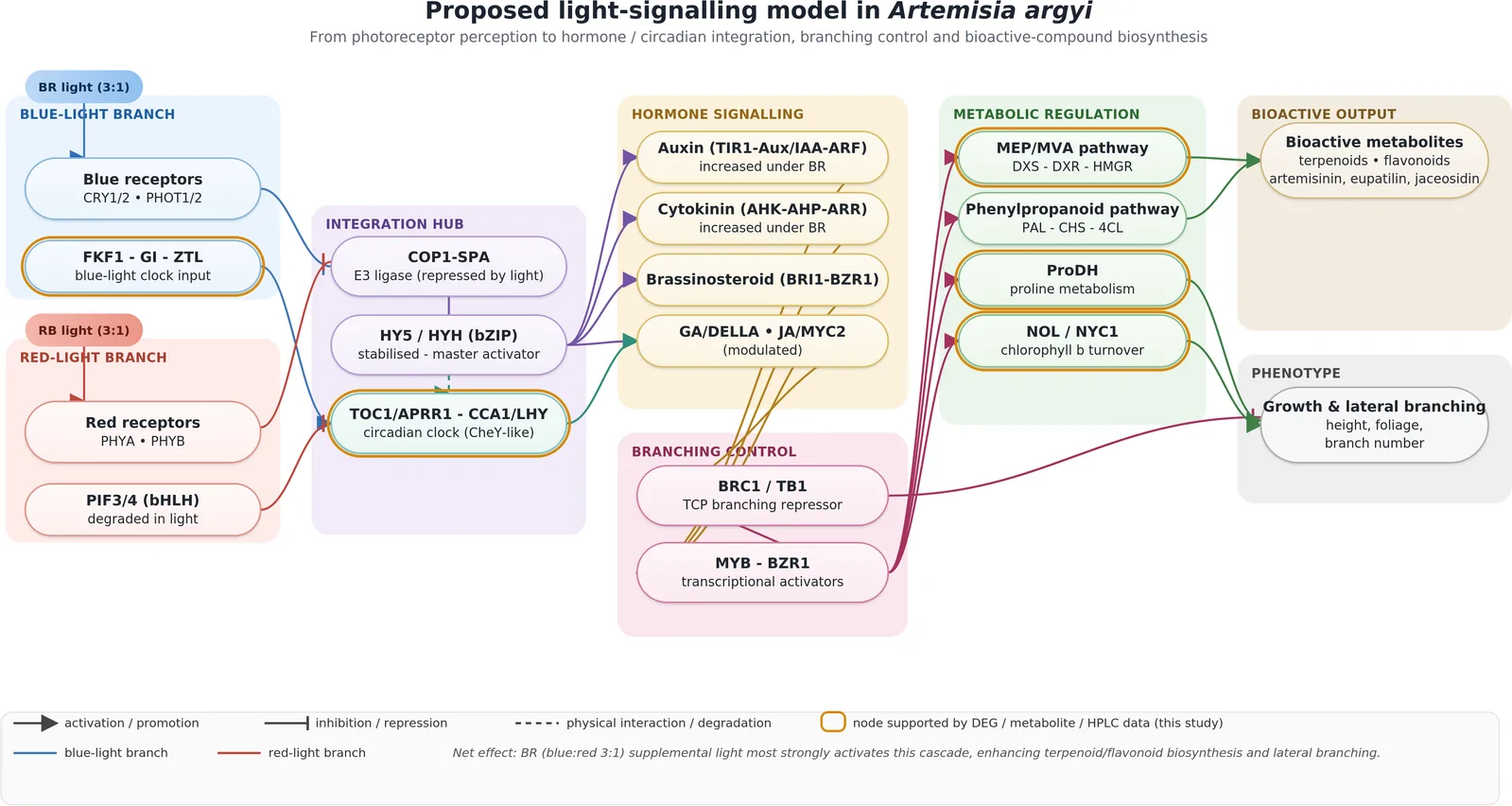
Proposed light-signaling model in *Artemisia argyi*. Blue- and red-light photoreceptors converge on the COP1–SPA/HY5 hub and the circadian clock (TOC1/APRR1, FKF1–GI–ZTL, identified in this study); stabilized HY5 activates auxin, cytokinin, and brassinosteroid signaling, which together repress the branching regulator BRC1/TB1 and, via MYB/BZR1, activate the MEP/MVA and phenylpropanoid biosynthetic genes, ProDH and NOL/NYC1, linking the photoreceptor signal to the terpenoid and flavonoid bioactives and the enhanced lateral branching that accumulates under blue-enriched (BR) light. Nodes outlined in gold were supported directly by the DEG, metabolite, or HPLC data generated in this study.

A current limitation of this study is that regulation was inferred from transcriptomic and metabolomic data without a matching proteome; thus, post-transcriptional and post-translational control of the pathway cannot be directly confirmed. Comparative quantitative proteomics (e.g., TMT-based LC-MS/MS) across the BR, RB, and white-light treatments would allow the protein-level abundance of the photoreceptor, circadian, hormone-signaling, and biosynthetic enzymes highlighted in **Figure 12** to be measured directly; test whether transcript-level differences are preserved at the protein level; and strengthen the proposed protein–protein interaction model with empirical abundance and interaction data. We identify this, together with side-by-side monochromatic red- and blue-only light treatments, as a priority for follow-up work.

## Conclusions

Collectively, our findings highlight the multifaceted role of blue light in regulating growth, metabolism, and stress adaptation in *A. argyi*. Among the tested light treatments, the BR combination was most effective in promoting vegetative development, activating metabolic pathways, and enhancing the accumulation of bioactive compounds. Among them, CheY-like phosphoacceptor encoded by the two-component response regulator-like APRR1, proline dehydrogenase, and the chlorophyll b reductase NOL catalyzing the degradation of chlorophyll b and isoprene genes were the most promising findings. These findings offer valuable insights into light regimes for medicinal plant cultivation and improving phytochemical content. Moreover, further studies are needed to assess the long-term effects of supplemental light exposure on the sustained production of secondary metabolites and to explore their implications for commercial-scale cultivation and pharmaceutical application.

## Data Availability

The data presented in the study are deposited in the Zenodo repository, accession number https://doi.org/10.5281/zenodo.22828151.
